# The Expression of TALEN before Fertilization Provides a Rapid Knock-Out Phenotype in *Xenopus laevis* Founder Embryos

**DOI:** 10.1371/journal.pone.0142946

**Published:** 2015-11-18

**Authors:** Kei Miyamoto, Ken-ichi T. Suzuki, Miyuki Suzuki, Yuto Sakane, Tetsushi Sakuma, Sarah Herberg, Angela Simeone, David Simpson, Jerome Jullien, Takashi Yamamoto, J. B. Gurdon

**Affiliations:** 1 Wellcome Trust/Cancer Research UK Gurdon Institute, University of Cambridge, Cambridge, United Kingdom; 2 Department of Mathematical and Life Sciences, Graduate School of Science, Hiroshima University, Higashi-Hiroshima, Japan; National University of Singapore, SINGAPORE

## Abstract

Recent advances in genome editing using programmable nucleases have revolutionized gene targeting in various organisms. Successful gene knock-out has been shown in *Xenopus*, a widely used model organism, although a system enabling less mosaic knock-out in founder embryos (F0) needs to be explored in order to judge phenotypes in the F0 generation. Here, we injected modified highly active transcription activator-like effector nuclease (TALEN) mRNA to oocytes at the germinal vesicle (GV) stage, followed by *in vitro* maturation and intracytoplasmic sperm injection, to achieve a full knock-out in F0 embryos. Unlike conventional injection methods to fertilized embryos, the injection of TALEN mRNA into GV oocytes allows expression of nucleases before fertilization, enabling them to work from an earlier stage. Using this procedure, most of developed embryos showed full knock-out phenotypes of the pigmentation gene *tyrosinase* and/or embryonic lethal gene *pax6* in the founder generation. In addition, our method permitted a large 1 kb deletion. Thus, we describe nearly complete gene knock-out phenotypes in *Xenopus laevis* F0 embryos. The presented method will help to accelerate the production of knock-out frogs since we can bypass an extra generation of about 1 year in *Xenopus laevis*. Meantime, our method provides a unique opportunity to rapidly test the developmental effects of disrupting those genes that do not permit growth to an adult able to reproduce. In addition, the protocol shown here is considerably less invasive than the previously used host transfer since our protocol does not require surgery. The experimental scheme presented is potentially applicable to other organisms such as mammals and fish to resolve common issues of mosaicism in founders.

## Introduction

Programmable nucleases such as TALENs and CRISPR/Cas9 have been used to efficiently produce gene knock-out animals in various species including those previously regarded as non-permissive by conventional gene disruption methods [1–9]. *Xenopus* is one of the model organisms that is widely used in studying developmental biology, cell biology and nuclear reprogramming [10], and hence a sophistication of genome editing technologies in *Xenopus* is very desirable. Zinc finger nucleases were originally used to disrupt genes in *Xenopus tropicalis* embryos [11]. Notably TALENs-mediated genome editing has been shown to be a convincing route to achieve gene knock-out in *Xenopus tropicalis* and *Xenopus laevis* although the resulting knock-out embryos often exhibit a mosaic of mutant and wild-type phenotypes in founders (F0) [12–16]. Gene knock-out in *Xenopus laevis* is supposed to be more challenging than that in *Xenopus tropicalis* due to the allotetraploid genome although *Xenopus laevis* has unique characteristics such as larger oocytes, extensively characterized egg extracts and established reprogramming assays [17,18].

TALENs consist of a customized DNA-binding domain and a nuclease domain derived from Fok I endonuclease. A pair of customized TALENs (right and left side TALENs) binds to their target sites and then Fok I is dimerized to introduce DNA double strand breaks. This is followed by DNA repair mainly mediated by non-homologus end joining, thus resulting in deletion and/or insertion mutations in targeted genes. Such TALEN activity has recently been enhanced by modifying repeat sequences in the TALE DNA-binding modules, referred to as Platinum TALEN [15]. Moreover, it has recently been shown that Platinum TALEN fits well with the efficient gene knock-in system based on microhomology-mediated end-joining at the TALEN-targeted site [19].

Several genes have been knocked out using TALENs in *Xenopus* [12–14]. *tyrosinase* (*tyr*), a gene involved in melanin synthesis, is one of the most targeted genes to discern the effect of TALENs since the resulting knock-out frogs show the albino phenotype. Embryonic lethal genes have also been disrupted by TALENs. *pax6* knock-out by TALEN mRNA injection to one-cell stage embryos results in eye deformation in F0 embryos [12,20], and null mutants for this gene die at the tadpole stage due to axial defects [20]. In addition to the disruption of TALEN-targeted short sequences, two sets of TALENs have been used to cut out a large genetic locus in *Xenopus tropicalis pax6*, resulting in 10 kb deletion [20]. In theory this approach is useful for disrupting long non-coding sequences such as promoters/enhancers and non-coding RNA [21].

In this study, we used Platinum TALENs to establish a less-mosaic gene knock-out system in *Xenopus laevis* to observe full knock-out phenotypes in F0 embryos. We first hypothesized that mosaicism of F0 embryos is attributable to the injection of TALEN mRNAs into fertilized embryos since injected mRNAs are not readily distributed evenly throughout embryos by the time when the embryo has already divided into several cells. Therefore, we have performed the injection of TALEN mRNAs into *Xenopus* oocytes at the germinal vesicle (GV) stage by taking advantage of the oocyte injection and culture system [18,22,23]. By this means, TALE nucleases are expressed before fertilization, and maternal and paternal genomes are exposed to TALENs for a much longer time than when they are injected into fertilized embryos. This experimental setup enabled multiple gene disruption up to 100% efficiency and allowed us to obtain founder embryos with full knock-out phenotypes.

## Materials and Methods

### Animals

All experimentation with frogs was carried out following requirements of the UK Home Office and following the guidelines of Hiroshima University for the care and use of experimental animals. This research project was approved by the UK Home Office (licence reference number: PPL80/2548) and by the committee of Hiroshima University for the use and care of experimental animals (approval number: G13-2). For collecting oocytes, frogs were anesthetized by subcutaneous injection of 120 mg (in 400 μl) of Tricaine methanesulfonate (MS222). Subsequently, the frogs were slaughtered by exsanguination under anesthesia, followed by freezing for appropriate disposal. Importantly, our refined protocol does not require frog surgery unlike the previously published host transfer method [24], thereby addressing the Home Office Three R’s guidelines (Replacement, Reduction and Refinement).

### Oocyte preparation, TALEN mRNA injection, *in vitro* maturation and ICSI

Injection to immature oocytes, oocyte maturation and intracytoplasmic sperm injection (ICSI) were performed by following our previously published protocol [18]. Moreover, a very detailed protocol with video instruction for this method has recently been published [23]. A change to this published protocol is that TALEN mRNAs were injected instead of antisense oligonucleotides. A brief protocol with the minor change incorporated is shown below.

Oocytes were collected from frogs injected with pregnant mare's serum gonadotropin (PMSG) and defolliculated using liberase (Roche). Oocytes were injected with 250 pg each of right and left TALEN mRNAs (*tyr* and *pax6*) and cultured in Modified Barth’s Solution (MBS) with 0.1% BSA at 18°C. For *mars2-l* knock-out experiments, two sets of right and left TALEN mRNA pairs were injected. Control oocytes were injected with 500 pg of right TALEN mRNA only. After a few hours incubation, TALEN mRNA-injected oocytes were transferred into MBS with 3 μM progesterone. After overnight treatment with progesterone (16 h at 16°C), progesterone-containing solution was washed away and *in vitro* matured oocytes were moved onto agarose-coated plates filled with the injection solution [23]. *In vitro* matured eggs were then subjected to ICSI using a frozen sperm stock diluted with the sperm dilution buffer [23]. Embryonic cleavage was examined 5–6 h after ICSI and cleaved embryos were transferred to the incubation solution [23]. Next morning, surviving embryos were transferred to 0.1x Marc’s Modified Ringer (MMR) and further incubated.

Injection to fertilized one-cell stage embryos was performed by following our previous reports [12,18].

### Construction of TALEN plasmids

A two-step Golden Gate assembly method using the Platinum Gate TALEN Kit (Addgene; cat#1000000043) [15] was used to construct Platinum TALEN plasmids containing the homodimer-type FokI nuclease domain. The assembled repeat arrays were subsequently inserted into the final destination vector, ptCMV-153/47-VR. For double knock-out experiments, previously reported TALENs for *pax6* were used [12]. Target sequences for TALENs are summarized in S1 Table.

### Identification of *mars2-like* gene

The transcript sequence of *mars2-like* (*mars2-l*) was originally found in transcriptome assembly (accession ID: SRA279316), which was mapped to the *Xenopus laevis* genome 6.1 [18,25]. The *mars2-like* sequence is also found in *Xenopus laevis* 6.0 genome using BLAST (http://www.xenbase.org/genomes/blast.do). The scaffold position of the exon that was knocked out in this study is at Scaffold189860:120472.121801 (+ strand) and the parent_id is MARS2|Horb201201_XENLA_00089909|JGIv6.000189860_120471_12393 2|f1 in *Xenopus laevis* 6.0 genome. The *mars2-l* map including exon information was obtained from *Xenopus laevis* 6.1 genome. The *mars2-l* sequence was aligned with the *mars2* sequence (NCBI: NM_001086369.1). *mars2-l* does not seem to encode a meaningful protein in any frames, although it carries 87% sequence homology to *mars2*.

### Genotyping of embryos

TALENs-expressed or control embryos at the indicated stages were collected and at least 3 sets of a single embryo in each treatment were transferred into Eppendorf tubes. DNA was extracted using DNeasy Blood and Tissue kit (QIAGEN). For knock-out analyses of *mars2*, one exon of *mars2-l* or the corresponding exon of *mars2* was amplified using specific PCR or qPCR primers. Importantly, primers for *mars2-l* do not recognize *mars2*, and vice versa. qPCR data were normalized by using an untargeted *hox* gene (*hoxb1*). For genotyping of *tyr* knock-out embryos, extracted DNA from a single embryo was subjected to genomic PCR using homeolog-specific primer sets. PCR products were subcloned into pCR2.1/TOPO (Life Technologies) by TA cloning. Colony PCR was performed to select positive clones, which were then subjected to DNA sequencing. Restriction Fragment Length Polymorphism (RFLP) analyses of *tyr* and *pax6* were performed using HinfI (TaKaRa) or Hpy188I (NEB), respectively [12,16].

Primers used are summarized in S1 Table.

### Statistical tests

Numbers of experimental replicates are shown as N. For *mars2-l* knock-out assays, statistical differences were calculated by two-tailed F- and T-test. The levels of significance were set as * P < 0.05 and ** P < 0.01. Error bars were represented as the standard error of the mean.

## Results

### The full knock-out phenotype after disrupting the *tyrosinase* gene in *Xenopus laevis* founder embryos

**Fig 1:**
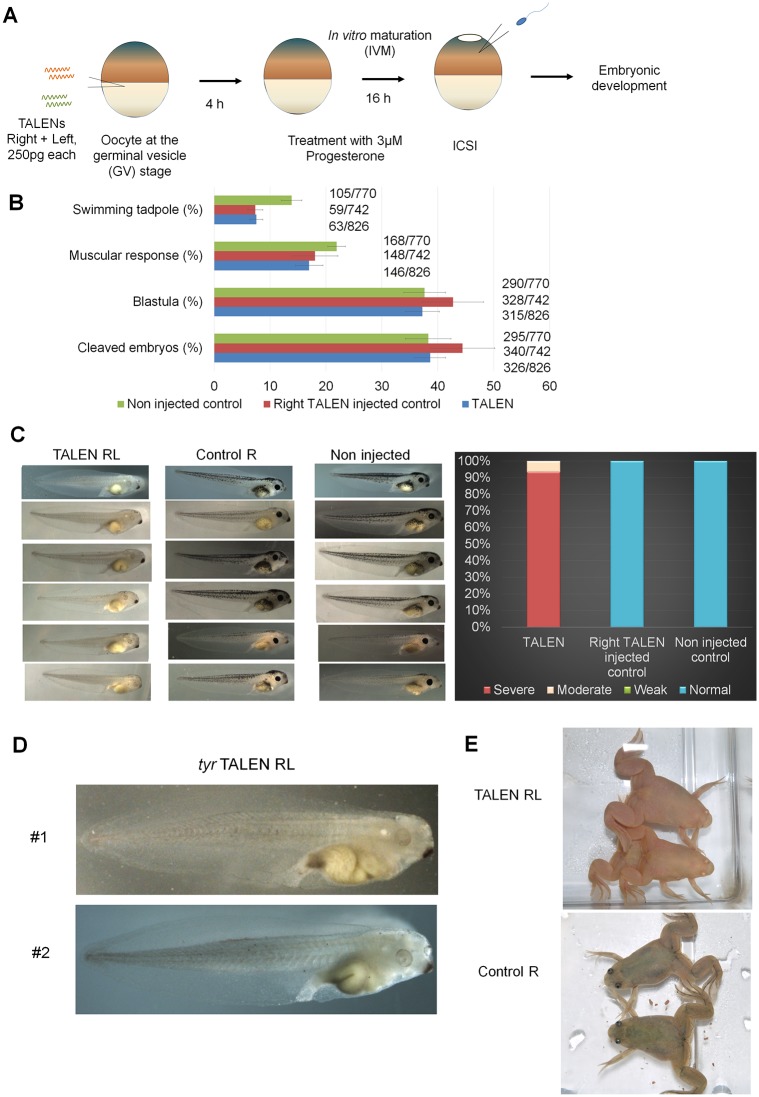
Injection of TALEN mRNAs into GV oocytes allows efficient *tyrosinase* gene disruption. (A) Schematic diagram of the experiment to express TALENs in *Xenopus laevis* eggs before fertilization. (B) Development of *tyrosinase* (*tyr*) TALENs-expressed oocytes. TALEN mRNA-injected GV oocytes, followed by *in vitro* maturation and ICSI, were able to develop to the swimming tadpole stage although mRNA injection itself seems to decrease the developmental capacity of *in vitro* matured eggs. Actual numbers of embryos that were injected with sperm and that developed to each developmental stages are indicated next to the corresponding bars. (C-E) Expression of *tyr* TALENs in GV oocytes allowed almost complete albino phenotypes in F0 embryos and frogs. Enlarged pictures of two TALEN-RL-expressed tadpoles are shown in Fig 1D. Control R represents control embryos and frogs in which only right side TALEN was expressed.

TALEN mRNAs against *Xenopus laevis tyrosinase* (*tyr*) were injected into GV oocytes (Fig 1A). The injected oocytes were *in vitro* matured and fertilized by intracytoplasmic sperm injection (ICSI) (Fig 1A). Sperm was directly delivered into an egg by ICSI because *in vitro* matured eggs do not carry the jelly layer, whose components are needed for the *in vitro* fertilization by incoming sperm. Since *Xenopus laevis* carries allotetraploid genome, disruption of all four genomic alleles of *tyra* and *tyrb* on both maternal and paternal genome is needed to observe the full albino phenotype [26]. Sixty-three swimming tadpoles were obtained out of 326 cleaved embryos in both right and left side TALEN-expressed embryos (TALEN-RL) in 4 independent injection experiments (Fig 1B, blue bar). Tadpoles were classified into 4 groups depending on the loss of pigmentation in the retinal pigment epithelium, severe, moderate, weak and normal (unchanged) according to the previously reported criteria [12,16]. Remarkably, 93% of tadpoles exhibited the severe knock-out phenotype (Fig 1C and 1D), suggesting TALENs were able to simultaneously induce mutations even in the allotetraploid genome. Embryos expressed with right side TALENs (Control R) and non-injected embryos showed normal pigmentation (Fig 1C). Finally, 8 mutant tadpoles went through metamorphosis and all showed nearly complete albino phenotype (Fig 1E). DNA sequencing analyses of all four alleles revealed that approximately 90% of *tyra* and *tyrb* loci in TALEN-expressed tadpoles were mutated (S1 Fig). A few different types of mutations were found in the *tyra* gene (S1 Fig), suggesting that mutations happen at relative early stages in embryonic development. These results suggest that the injection of *tyr* TALEN mRNAs into GV oocytes enables the full albino phenotype in the F0 generation.

### Knock-out of an embryonic lethal gene in the F0 generation

**Fig 2:**
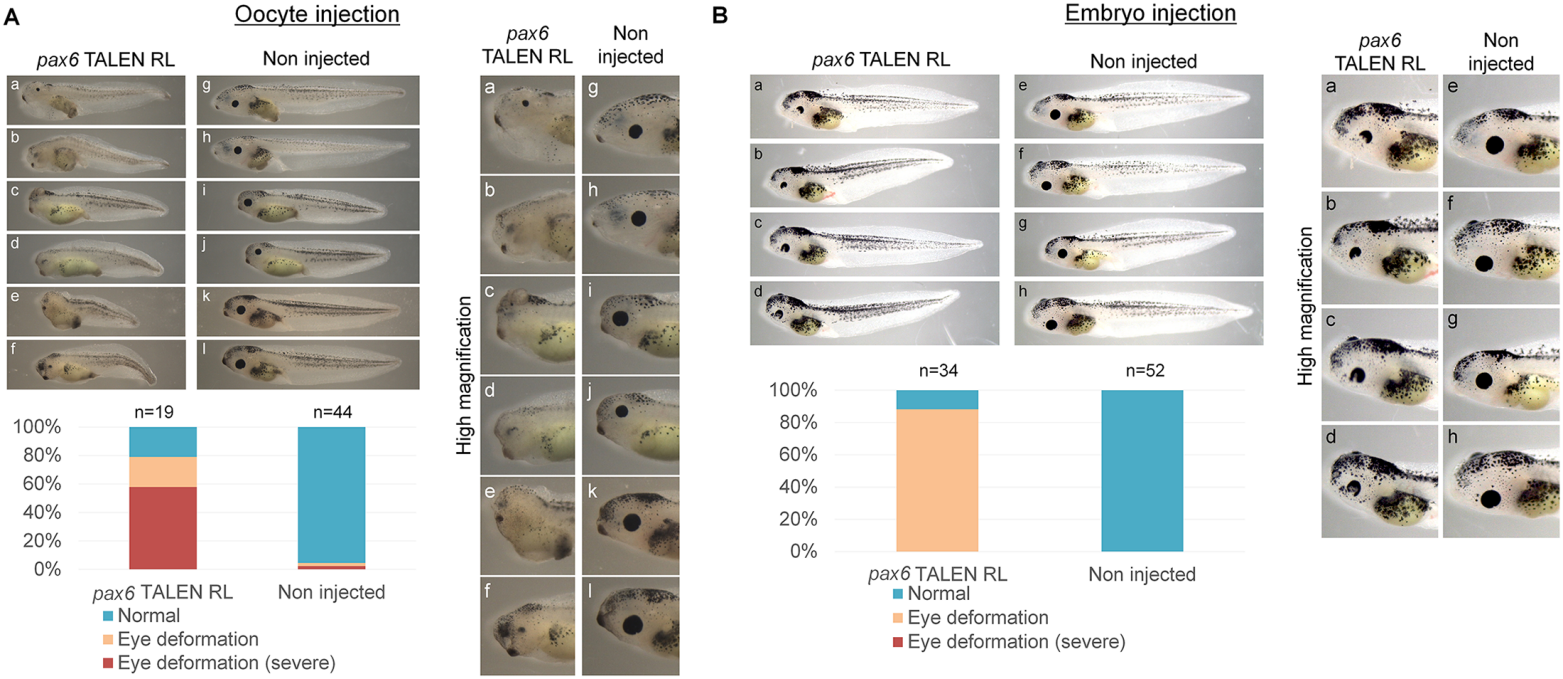
Phenotypes of embryos that were injected with *pax6* TALEN mRNAs. (A) Injection of *pax6* TALEN mRNAs to GV oocytes, followed by *in vitro* maturation and ICSI, recapitulated the null mutant phenotype of *pax6* in F0 embryos. Examples of knock-out tadpoles with different magnifications (a-f; *pax6* TALEN-expressed tadpoles, g-l; control tadpoles) are shown. The percentages of embryos that showed the knock-out phenotype of *pax6* are summarized in the graph, as judged by the degree of eye deformation. Embryos without TALEN mRNA injection are used as a control (Non injected). (B) Different phenotypes are observed at the tadpole stage between conventional embryo injection (Fig 2B) and the oocyte injection method (Fig 2A). TALEN mRNAs were injected into fertilized one-cell stage embryos. Examples of *pax6* TALEN-expressed tadpoles with different magnifications (a-d; *pax6* TALEN-expressed tadpoles, e-h; control tadpoles) are shown.

**Fig 3:**
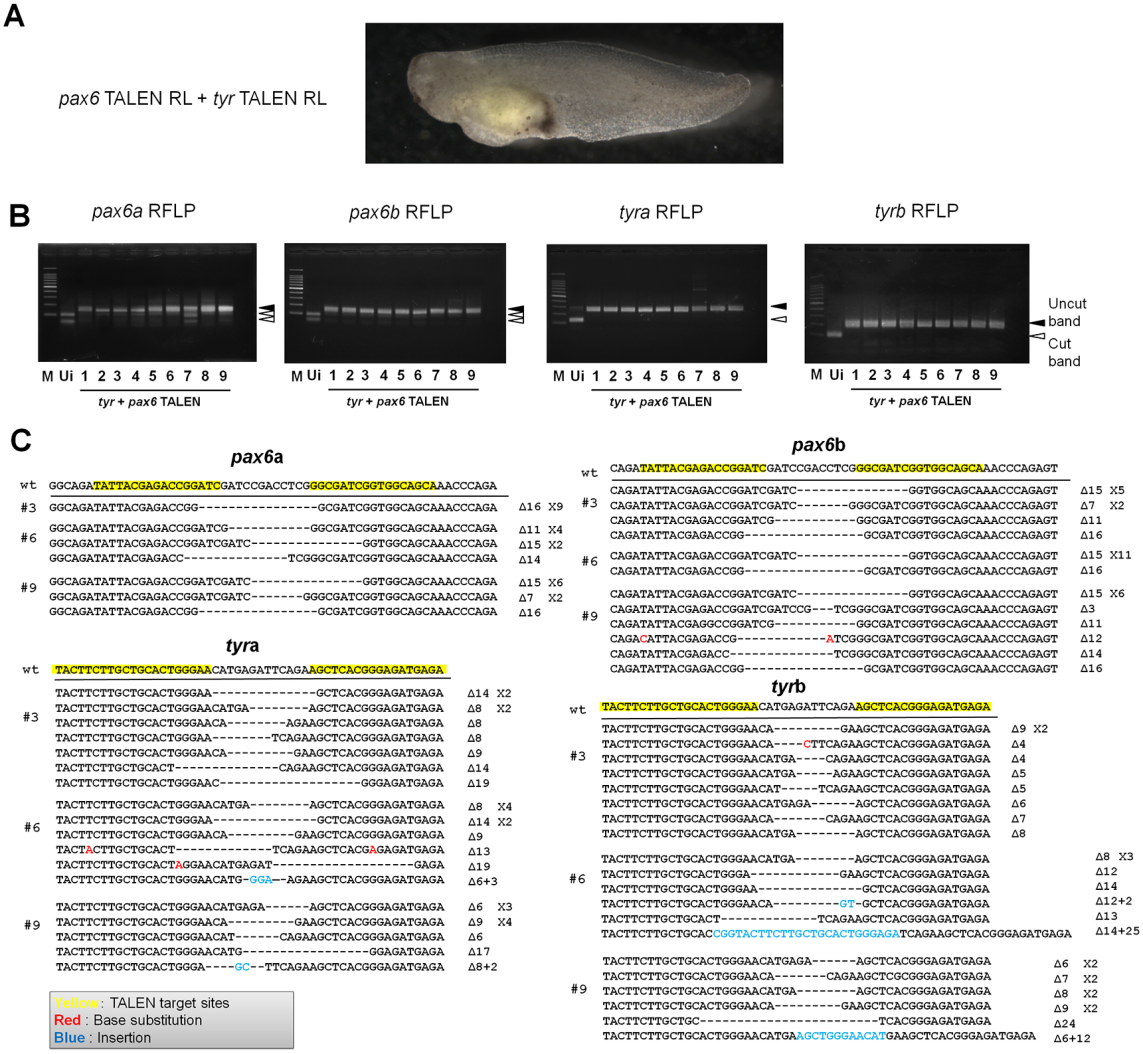
Double knock-out of *pax6* and *tyr* in *Xenopus* F0 embryos. (A) Double knock-out of *pax6* and *tyr* was achieved by TALEN mRNA injection to GV oocytes. A double knock-out tadpole is shown. (B) Detection of *pax6* and *tyr* mutants by RFLP analysis. Wild type embryos are cut by restriction enzymes (Ui: uninjected embryo), while mutant embryos are not digested. Digested PCR products appear at lower bands (marked by open arrowheads) and the undigested is marked by closed arrowheads. M represents 100 bp ladder marker. (C) Sequencing of three different embryos (#3, #6, #9; the numbers correspond to those in Fig 3B) revealed the 100% mutation rate in *pax6* and *tyr* double knock-out embryos.

The full knock-out phenotype after *tyr* targeting prompted us to test whether an embryonic lethal phenotype can be recapitulated in the F0 generation by disrupting such a gene using our strategy. We selected *pax6* as a target because the null mutant *Xenopus* embryos of *pax6* die at the swimming tadpole stage [20] and because the phenotype of *pax6* knock-out or knock-down has been characterized [12,20,27]. We designed TALENs that recognize the paired domain in exon 6 of both *pax6* homeologs, *pax6a* and *pax6b*. The *pax6* TALEN mRNAs were injected into oocytes, and the injected oocytes were *in vitro* matured and fertilized as described (Fig 1A). An eighty percent of swimming tadpoles showed the *pax6* mutant phenotype; deformed eyes or small eyes (Fig 2A). Furthermore, severe knock-out embryos (60% of all tadpoles) also showed delayed development of digestive tract [27] and axial defects, which are observed in *pax6* null mutant embryos [20]. Interestingly, these severe mutant phenotypes were not seen when *pax6* TALEN mRNAs were injected into one-cell stage embryos (Fig 2B), suggesting that oocyte injection allows the stronger knock-out phenotype. We then tried double knock-outs of *pax6* and *tyr*. The tadpoles obtained showed almost complete loss of pigmentation in eyes and on the trunk region (Fig 3A). The loss of pigmentation in eyes made it difficult to judge *pax6* mutant phenotypes, but Restriction Fragment Length Polymorphism (RFLP) analysis confirmed successful mutagenesis in all 9 tadpoles examined (Fig 3B). Furthermore, DNA sequencing analysis of double knock-out embryos revealed that 3 out of 3 embryos examined showed mutations in 100% of sequenced alleles (Fig 3C). In conclusion the oocyte injection method enables us to evaluate an embryonic lethal phenotype in founder embryos. Our protocol also allows multiple knock-outs simultaneously.

### Two sets of TALENs allow a deletion of long non-coding sequences

**Fig 4:**
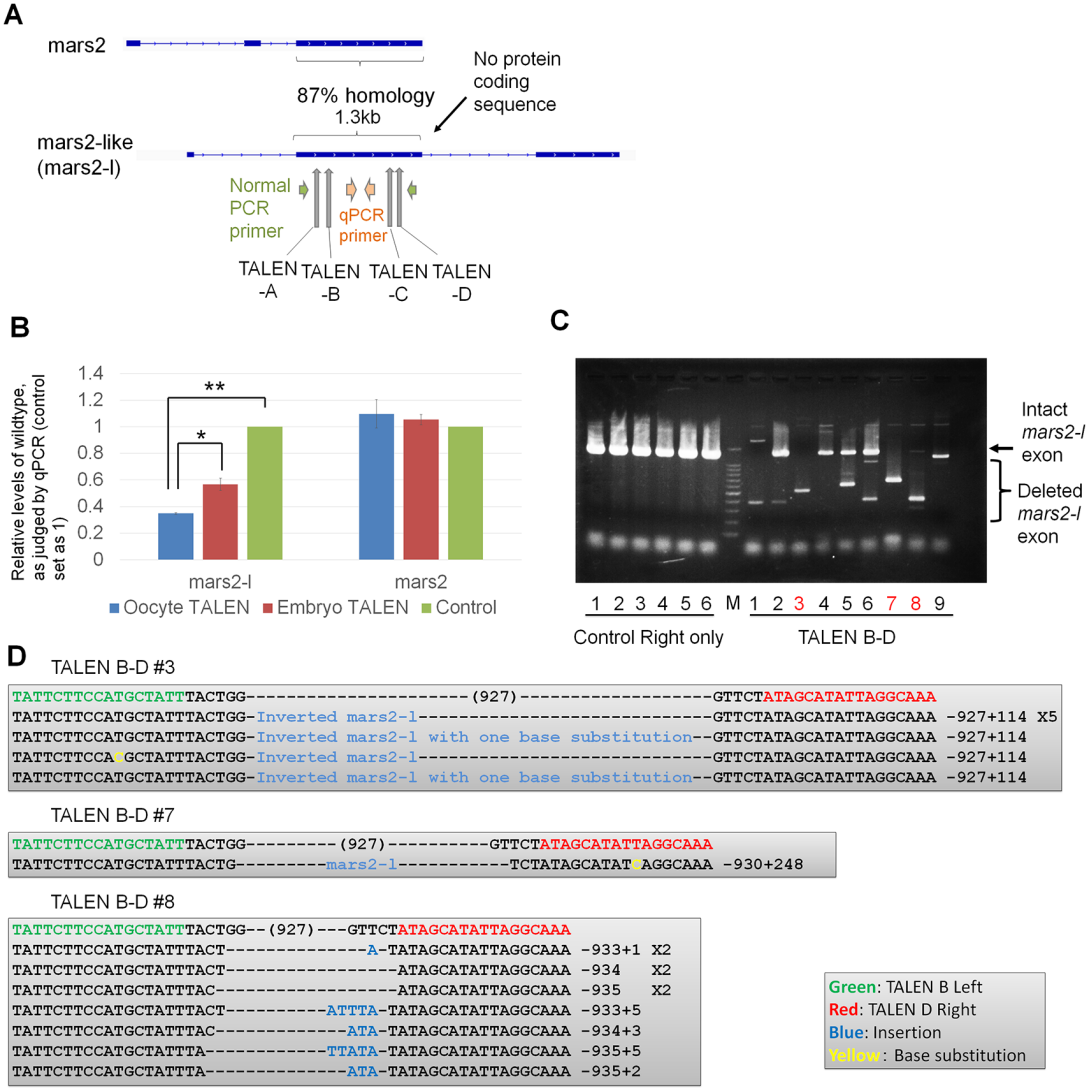
The deletion of a large genetic locus is achieved by the expression of two sets of TALENs by oocyte injection. (A) TALENs were designed to disrupt one of the exons of *mars2*-like (*mars2-l*) gene. PCR primers were used to confirm gene knock-out. Exons are marked by boxes. (B) The injection of TALEN mRNAs into GV oocytes (blue bar), but not into fertilized embryos (red bar), showed better knock-out of the *mars2-l* exon, as revealed by qPCR analyses. Control represents embryos injected only with right TALEN mRNA. N = 3–4 independent experiments. Error bars are standard errors. *P < 0.05. **P < 0.01. (C) Expression of TALENs before fertilization allowed the production of an embryo carrying only the deleted *mars2-l* exon in the F0 generation (lane 3, 7 and 8 in TALEN B-D, red color). Genomic DNA was extracted from single embryos at St. 10.5–11 and subjected to PCR analysis. M represents 1.5 kp and 100 bp ladder marker. (D) Large deletions of exon 2 in *mars-l* were confirmed by sequencing. Sample numbers correspond to those in Fig 4C. A part of inverted *mars-l* sequence was inserted in sample #3.

We next asked if long non-coding sequences can be deleted using our strategy (Fig 1A). In order to judge off target effects, we chose a non-coding sequence that has high homology to a protein coding gene. During bioinformatic searches for transcripts upregulated after the midblastula transition, we found a transcript that carries 87% sequence similarity with an exon of *mars2* (NCBI: NM_001086369.1), but does not seem to encode any proteins (Fig 4A) [28]. Hereafter this transcript is referred to as *mars2*-like (*mars2-l*, see Materials and Methods). We designed four sets of TALEN pairs spanning an exon of the *mars2-l* gene (TALENs-A and –B at the beginning of exon 2, TALENs-C and -D at the end of exon 2; Fig 4A and S2 Fig). Different combinations of two TALEN pairs (A-C, A-D, B-C and B-D) were expressed by mRNA injection to one-cell stage embryos (S3A Fig). While all combinations of TALEN pairs allowed deletion of an almost entire exon of *mars2-l* in a part of embryos, the combination of TALENs B and D showed partial deletion in all samples examined. The off target effect on *mars2* was not observed (S3A Fig). However, we did not get embryos that have a tetraallelic knock-out of the exon by zygote injection (S3A Fig). In contrast, when TALEN mRNAs (TALEN-B and -D) were injected into oocytes, followed by *in vitro* maturation and ICSI (S3B Fig), rates of deleting the exon were significantly improved as measured by qPCR analyses (P < 0.05, Fig 4B). Importantly, embryos that carry only mutated exon were obtained by this method (Fig 4C, Red letters). The presence of single major mutated bands in lanes 3, 7 and 8 suggests that the same type of deletion was introduced on all alleles at least in these samples (Fig 4C). Sequencing analysis of target sites confirmed exon deletion or the insertion of *mars2-l* sequences (Fig 4D). Off target effects on *mars2* were not observed (S3C Fig). Together, the expression of two sets of TALEN pairs by oocyte injection enables the deletion of long non-coding sequences in F0 embryos.

## Discussion

We here report a method to achieve full knock-out phenotypes in *Xenopus laevis* F0 embryos. The presence of untargeted wild type cells in F0 genome-edited embryos has been an issue after injection of programmable nucleases to one-cell stage embryos. The injection of TALEN mRNAs from the GV oocyte stage reduced unedited cells in F0 embryos as judged by *tyr* knock-out, and the embryonic lethal phenotype of *pax6* knock-out was recapitulated in most of F0 embryos. These strong knock-out phenotypes seem to be caused by the presence of TALEN proteins by the time of fertilization, which then allows instant access to zygotic chromatin and to chromatin of embryos at early cleavage stages.

Although the protocol shown here is technically demanding compared to the conventional zygote injection, it instead offers a unique opportunity to obtain embryos showing full knock-out phenotypes in the F0 generation. It is also noteworthy that our strategy allows the disruption of a 1 kb genomic locus, as shown by *mars2-l* deletion in F0 embryos. Although the identity of *mars2-l* is not defined in this study, *mars2-l* knock-out experiments in theory suggest that our method can be used to disrupt long non-coding RNA without off target effects. The sequencing analyses of those knocked out embryos revealed several different mutation patterns in F0 embryos, implying that TALEN-mediated gene disruption in our system may start to work in early embryos, but not in oocytes. This result is in good agreement with the lack of non-homologus end joining activity in oocyte nuclei [29]. Alternatively, TALENs may keep cutting the target site even after the initial action [30]. In any cases, the observed stronger knock-out phenotypes by the oocyte injection suggest that more abundant and/or evenly distributed TALEN proteins by oocyte injection results in a better knock-out. Considering that frogs normally take more than a year to sexual maturity, gene-manipulated frogs derived from the oocyte injection can be regarded as a rapid route to obtain frogs with full knock-out phenotypes without waiting for the next generation. Meantime, it is still necessary to perform multi-generation experiments to obtain embryos that carry the same mutant alleles in all cells since embryos produced in this study exhibited several different mutation patterns in F0 embryos, reminiscent of some degree of mosaicism. In addition to the rapid judgement of knock-out phenotypes, our experimental system may be applied for examining the effect of gene disruption on embryonic development at very early stages such as at the midblastula transition since TALENs work from earlier embryonic stages by the oocyte injection route than by the conventional zygote injection [24]. Therefore, our method can serve as a powerful tool for studying development, enabling phenotypic judgement of gene knock-out in *Xenopus laevis* F0 embryos.

In *Xenopus* research the oocyte host transfer technique, in which *in vitro* matured oocytes are transferred to an ovulating frog to coat eggs with jelly for fertilization, has been used to analyze functions of maternal or early zygotic genes such as beta-catenin by injecting antisense oligonucleotides and synthesized mRNA into GV oocytes [31,32]. The surgical transfer of oocytes to a recipient female frog can be omitted by directly delivering sperm into an *in vitro* matured egg [22]. Injection of mRNAs or antisense oligonucleotides into oocytes before *in vitro* maturation and ICSI can also be achieved [18,23] and TALEN mRNAs were injected in this report. Recently, the host transfer technique was used to fertilize oocytes that were injected with TALEN [24]. This paper also reports the perfect albino phenotype after targeting *tyr* in many of the treated embryos [24], in good agreement with our results. It is difficult to compare the efficiency of an entire process between the host transfer and the ICSI system since the development was not scored in the host transfer report [24]. However, our papers together support the contention that expression of programmable nucleases before fertilization results in full knock-out phenotypes. In the IVM-ICSI system, we can test several combinations of TALENs in one experiment. Thus, our described system may be applicable to a large scale analysis of gene targeting using programmable nucleases. Importantly, the IVM-ICSI method does not require frog surgery for transferring *in vitro* matured eggs, therefore tackling the 3R guidelines for animal care (Replacement, Reduction and Refinement).

Finally, other organisms that are used for the injection of nucleases into early embryos show mosaic knock-out phenotypes in the founder generations [33–35]. As far as the oocyte culture and maturation system is available, our strategy can be easily applied to other species. Since mammals often have established oocyte maturation systems [36], it is worth trying the injection of nucleases into immature oocytes. This is especially true of animals whose life cycle is long such as livestock and non-human primates. Moreover, the CRISPR/Cas9 system might also benefit from the oocyte injection because Cas9 protein is expressed before fertilization and because guide RNA can have a greater chance of gaining access to target sites. Our experimental scheme thus can potentially help the efficient production of genome-edited animals for agriculture and medical purposes.

## Supporting Information

S1 Fig Genotyping of *tyrosinase* TALENs-expressed embryos. Both *tyrosinase-a* (*tyra*) and *tyrosinase-b* (*tyrb*) showed approximately 90% mutation rates. Many deletion mutations show microhomologies at junctions (red underlines). The patterns of different mutations were less in *tyra* than in *tyrb*. This difference might be caused by recutting of target sites even after the initial non-homologus end joining although it is not clear why this happened preferentially in *tyrb*. DNA was extracted from a single embryo (tadpole) and genomic sequences containing target sites of TALENs were amplified by PCR. Primers specific for *tyra* or those for *tyrb* were used. Seven to 10 bacterial clones were picked up and sequenced from each tadpole.
(TIF)

S2 Fig TALEN target sequences for *mars2-l* knock-out. Sequences of *Xenopus laevis mars2-l* and *mars2* are aligned and target sequences of TALENs for *mars2-l* are marked by different colours. To delete an almost entire exon of *mars2-l*, each two sets of TALEN pairs were designed at the beginning (Mars2-l TALEN-A and –B) and at the end (Mars2-l TALEN-C and –D) of *mars2-l*.
(TIF)

S3 Fig Genotyping of *mars2-l* TALENs-expressed embryos. (A) Expression of two sets of TALEN pairs designed at the beginning and at the end of an exon of *mars2-l*, as shown in Fig 4A, resulted in deletion of the exon in a part of embryos while off-target effects on *mars2* were not observed. The combination of TALENs-B and -D showed reproducible knock-out. TALENs were injected into fertilized embryos. Each lane represents a result from a single embryo. (B) Development of TALEN mRNAs-injected oocytes, followed by *in vitro* maturation and sperm injection. Actual numbers of embryos that reached each developmental stages are indicated next to the corresponding bars. (C) Off-target effects of expressing TALENs B-D from the immature oocyte stage on *mars2* were not observed in embryos at Stage 11. As a control, only right TALENs were injected.
(TIF)

S1 Table TALEN target sequences and primer sequences. Red letters represent TALEN target sequences.
(DOC)
