# Supplementary figures and images for: The Expression of TALEN before Fertilization Provides a Rapid Knock-Out Phenotype in Xenopus laevis Founder Embryos
Source: PLoS One. 2015 Nov 18;10(11):e0142946. doi: 10.1371/journal.pone.0142946 (PMC4651567; doi:10.1371/journal.pone.0142946)

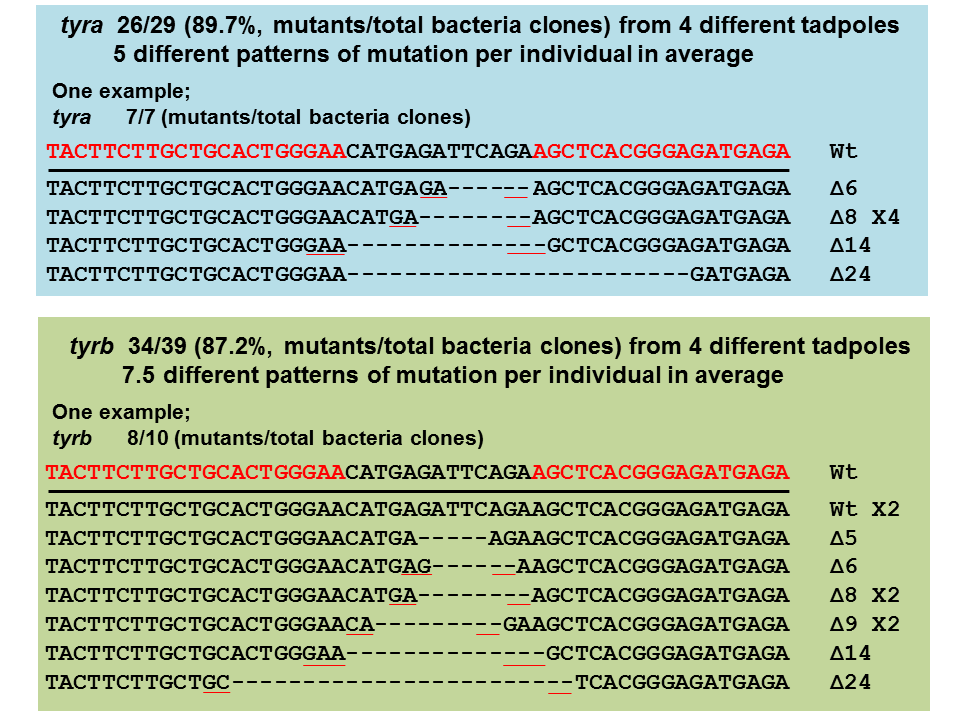

Supplement: S1 Fig — Both tyrosinase-a (tyra) and tyrosinase-b (tyrb) showed approximately 90% mutation rates. Many deletion mutations show microhomologies at junctions (red underlines). The patterns of different mutations were less in tyra than in tyrb. This difference might be caused by recutting of target sites even after the initial non-homologus end joining although it is not clear why this happened preferentially in tyrb. DNA was extracted from a single embryo (tadpole) and genomic sequences containing target sites of TALENs were amplified by PCR. Primers specific for tyra or those for tyrb were used. Seven to 10 bacterial clones were picked up and sequenced from each tadpole. (TIF) [file pone.0142946.s001.tif]

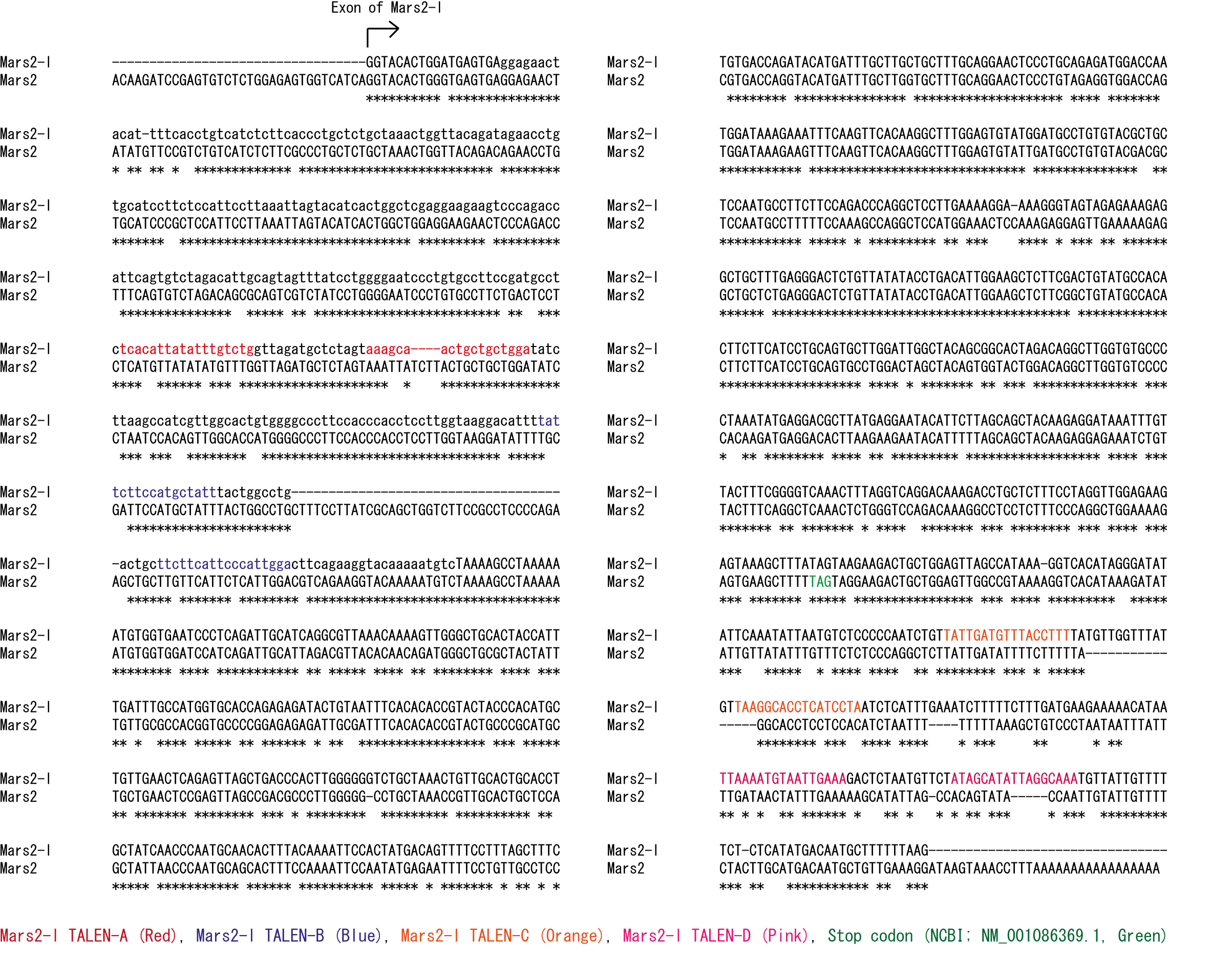

Supplement: S2 Fig — Sequences of Xenopus laevis mars2-l and mars2 are aligned and target sequences of TALENs for mars2-l are marked by different colours. To delete an almost entire exon of mars2-l, each two sets of TALEN pairs were designed at the beginning (Mars2-l TALEN-A and –B) and at the end (Mars2-l TALEN-C and –D) of mars2-l. (TIF) [file pone.0142946.s002.tif]

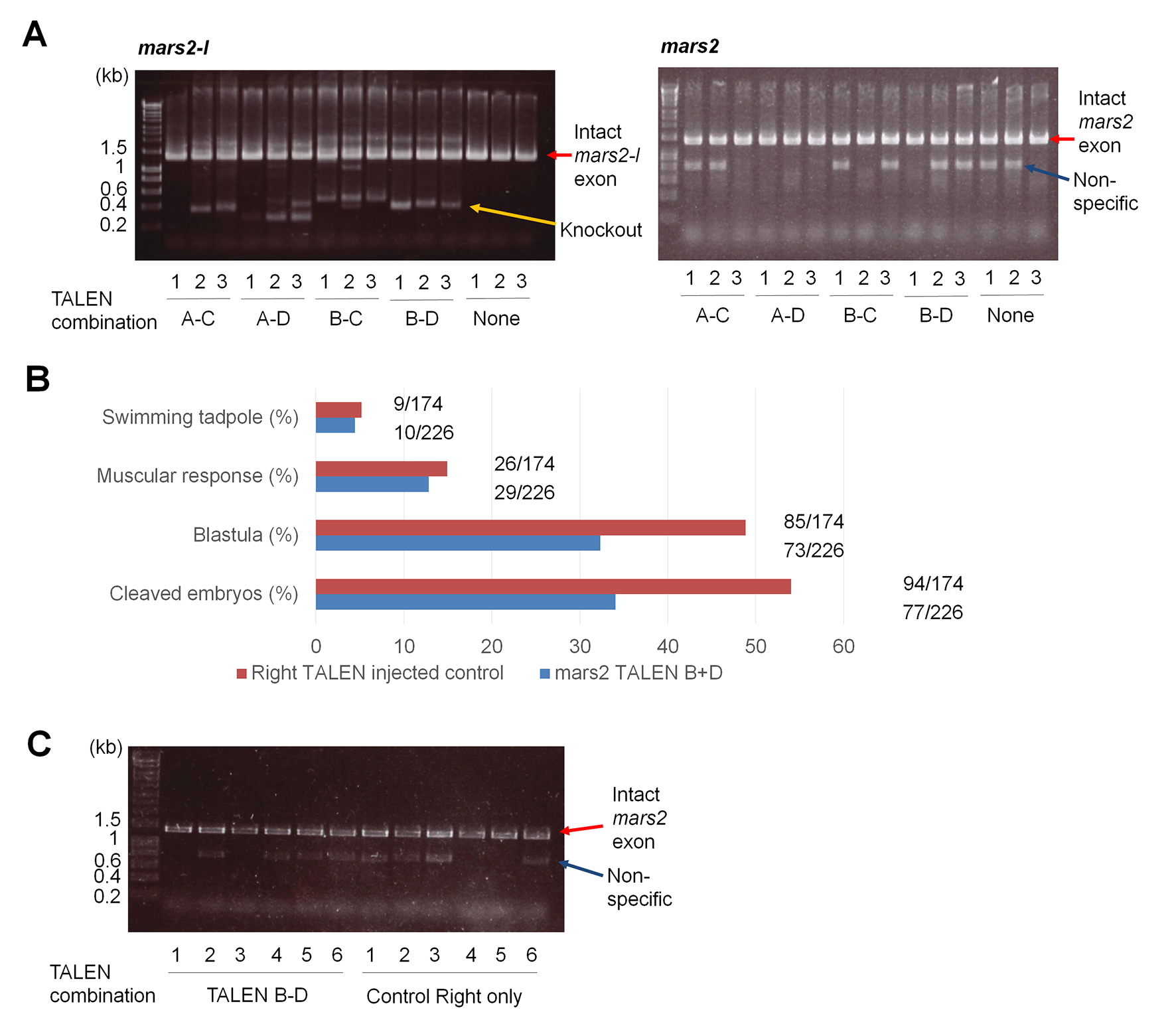

Supplement: S3 Fig — (A) Expression of two sets of TALEN pairs designed at the beginning and at the end of an exon of mars2-l, as shown in Fig 4A, resulted in deletion of the exon in a part of embryos while off-target effects on mars2 were not observed. The combination of TALENs-B and -D showed reproducible knock-out. TALENs were injected into fertilized embryos. Each lane represents a result from a single embryo. (B) Development of TALEN mRNAs-injected oocytes, followed by in vitro maturation and sperm injection. Actual numbers of embryos that reached each developmental stages are indicated next to the corresponding bars. (C) Off-target effects of expressing TALENs B-D from the immature oocyte stage on mars2 were not observed in embryos at Stage 11. As a control, only right TALENs were injected. (TIF) [file pone.0142946.s003.tif]
