# Supplementary material for: The Expression of TALEN before Fertilization Provides a Rapid Knock-Out Phenotype in Xenopus laevis Founder Embryos
Source: PLoS One. 2015 Nov 18;10(11):e0142946. doi: 10.1371/journal.pone.0142946 (PMC4651567; doi:10.1371/journal.pone.0142946)
Supplement: S1 Table — Red letters represent TALEN target sequences. (DOC) [file pone.0142946.s004.doc]

| S1 Table. TALEN target sequences and primer sequences. | | |
| --- | --- | --- |
| **A. TALEN target sequences** | |  |
|  | **Target sequence (5'→3')** | **References** |
| *tyr* TALEN (Platinum) | **tACTTCTTGCTGCACTGGGAACATGAGATTCAGAAGCTCACGGGAGATGAGa*** | Sakuma et al., 2013 |
| *pax6* TALEN | **tATTACGAGACCGGATCGATCCGACCTCGGGCGATCGGTGGCAGCa** | Suzuki et al., 2013 |
| *pax6* TALEN (Platinum) | **tATTACGAGACCGGATCGATCCGACCTCGGGCGATCGGTGGCAGCAAa** | In this study |
| *mars2-l* A TALEN (Platinum) | **tCACATTATATTTGTCTGGTTAGATGCTCTAGTAAAGCAACTGCTGCTGGa** | In this study |
| *mars2-l* B TALEN (Platinum) | **tATTCTTCCATGCTATTTACTGGCCTGACTGCTTCTTCATTCCCATTGGa** | In this study |
| *mars2-l* C TALEN (Platinum) | **tATTGATGTTTACCTTTTATGTTGGTTTATGTTAAGGCACCTCATCCTa** | In this study |
| *mars2-l* D TALEN (Platinum) | **tTAAAATGTAATTGAAAGACTCTAATGTTCTATAGCATATTAGGCAAa** | In this study |
| **B. Primer sequences.** | | |
|  | **Forward (5'→3')** | **Reverse (5'→3')** |
| *tyra* | CATCCCGAGATGCCTTCATAGGAG | GCTGGACTTAGACGGTTGCTCGT |
| *tyrb* | GGAGAGGATGGCCTCTGGAGAGATA | TGGTGGGATGGATTCCTCCCAGAAG |
| *pax6a* | CCAACGGCTGCGTCAGTAAGATCTT | CATGCAAAGATAGAAGGGCACTCTC |
| *pax6b* | CAACGGCTGCGTGAGTAAGATCTTG | GATAGAAGGGCACTCTCGCTTATAG |
| *mars2-l* normal PCR | GGTACACTGGATGAGTGAGG | GCATTGTCATATGAGAGAAAAAC |
| *mars2* normal PCR | GTACACTGGGTGAGTGAGGAG | TTTTACACTCACTTATTTTAAAGG |
| *mars2-l*  qPCR | TTGGCTACAGCGGCACTAGA | AATGTATTCCTCATAAGCGTCCTCAT |
| *mars2* qPCR | ACAAGGCTTTGGAGTGTATTGATG | AGCCTGGCTTTGGAAAAAGG |
| *hoxb1* qPCR | CCCGCCTTCCCTGCTT | AACCTTCCATCCCCATTATAAATG |

*Red letters represent TALEN target sequences.
